# The Emerging Role of Rab5 in Membrane Receptor Trafficking and Signaling Pathways

**DOI:** 10.1155/2020/4186308

**Published:** 2020-02-11

**Authors:** Wanqiong Yuan, Chunli Song

**Affiliations:** Department of Orthopedics, Peking University Third Hospital, 49 North Garden Rd., Haidian District, Beijing 100191, China

## Abstract

Ras analog in brain (Rab) proteins are small guanosine triphosphatases (GTPases) that belong to the Ras-like GTPase superfamily, and they can regulate vesicle trafficking. Rab proteins alternate between an activated (GTP-bound) state and an inactivated (GDP-bound) state. Early endosome marker Rab5 GTPase, a key member of the Rab family, plays a crucial role in endocytosis and membrane transport. The activated-state Rab5 recruits its effectors and regulates the internalization and trafficking of membrane receptors by regulating vesicle fusion and receptor sorting in the early endosomes. In this review, we summarize the role of small Rab GTPases Rab5 in membrane receptor trafficking and the activation of signaling pathways, such as Ras/MAPK and PI3K/Akt, which ultimately affect cell growth, apoptosis, tumorigenesis, and tumor development. This review may provide some insights for our future research and novel therapeutic targets for diseases.

## 1. Introduction

Ras analog in brain (Rab) proteins, belonging to the largest family of Ras superfamily, are small guanosine diphosphate (GTP)- bound proteins that regulate intracellular trafficking pathways [1]. There are more than 60 distinct proteins in humans, which constitute 41 functional subfamilies with tissue specificity. Rab proteins are similar to Ras and other GTP-bound proteins in their structures. They are composed of approximately 200 amino acids, and contain five highly conserved regions necessary for binding GTP and hydrolysis. Rab proteins are present in monomeric forms, and the amino acid sequence similarity of Rab family members ranges from 35% to 80% [2]. Rab proteins with more than 75% of sequence similarity can be identified as the same protein.

Rab5 is one of the most crucial members of the Rab family, whose functions and mechanisms are well studied. Rab5 transforms between the activated form, GTP-bound Rab5 (GTP-Rab5), and the inactivated form, guanosine diphosphate (GDP)- bound Rab5 (GDP-Rab5) [3]. Activated Rab5 interacts with its effectors and involves in vesicular transport, membrane trafficking, and signaling pathways [4].

In this review, we discussed the structure and activation of Rab5 and highlighted the recent advancements in the Rab5 regulating membrane receptor trafficking and signaling pathways, which will finally affect the occurrence and development of diseases.

## 2. The Rab GTPase Proteins

The Rab GTPase proteins were first studied in yeast *S. cerevisiae* by Novick. It was found a series of genes are necessary for the yeast secretion, which were named *SEC* (*SEC1, SEC2*, etc.) [5]. Subsequently, Gallwitz's group found the genes encoding the Ras-related *YPT1* protein in yeast *S. cerevisiae* [6]. Further studies showed that the mutants of both SEC4 and YPT1 could encode small GTP-bound proteins, and the structural and functional analogues of SEC and YPT were cloned from a rat brain library and named Rab [7].

Rab proteins share similar structures, generally containing two cysteine residues at the carboxyl terminus generally, which appear in the form of -CC, -CXC, -CCXX, -CXXX, or -CCXXX (X represents any amino acid) and act as the membrane localization signal [8]. The key structures of Rab GTPase proteins contain a highly conserved G domain that comprises six *β* sheets (*β*1–*β*6), five *α* helixes (*α*1–*α*5), and five polypeptide rings; N- and C-terminals; and the molecular switches I and II [2]. The N-terminus may be involved in isoprene modification of the C-terminal cysteine. Molecular switches I and II, and N- and C-terminals determine the function of Rab GTPase proteins together. Highly related Rab GTPase proteins may be expressed in the same organelle, but exert different functions.

**Figure 1:**
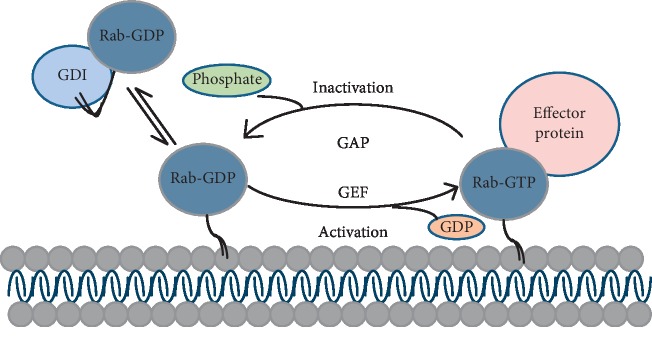
Transformation of Rab proteins between the activated and inactivated forms. GAP catalyzes the hydrolysis of GTP and inactivates the Rab proteins. GDI stabilizes GDP-Rab. GEF removes GDP via guanine exchange, allowing Rab binding to GTP and further interacting with downstream effectors.

Rab GTPases can transform between the GTP-bound activated form and GDP-bound inactivated form [8]. The GTP-Rab is located on the plasma membrane, and GDP-Rab is located in the cytoplasm. The transformation between the activated and inactivated forms requires three crucial regulators: GDP dissociation inhibitor (GDI), guanine nucleotide exchange factor (GEF), and GTPase activating protein (GAP). As shown in Figure 1, GDI as a circulating factor that regulates the binding and unloading of Rab GTPases on the plasma membrane. After being released by GDI, Rab is activated by GEF, which catalyzes the conversion of GDP to GTP. Then, Rab-GTP may perform its roles by recruiting the downstream effectors. The inactivation of Rab GTPases involves the following steps: GAP inactivates Rab GTPases by catalyzing the hydrolysis of GTP. GDI binds with inactivated Rab-GDP to form a complex, impeding the interaction between Rab proteins and their effectors. Then, inactivated Rab proteins are transferred from the plasma membrane into the cytoplasm to start a new cycle [9, 10]. Although with similar structure, Rab family proteins perform different functions in membrane receptor trafficking and signaling pathways because they bind to different effectors [4]. Rab GTPases play their roles in organelles connection at different stages of vesicular transport, including budding, transport, tethering, docking, and fusion stages [11].

## 3. The Basic Information of Rab5

Rab5 is a key member of the Rab family, and Rab5A is its most important subtype, with well-identified functions and mechanisms. Rab5 is mentioned as Rab5A in most studies. Rab5A is located at 3p24.3 and is composed of 215 amino acids with a molecular weight of 23.658 kD [12]. The protein structure of Rab5 is nearly spherical: *β* sheets and *α* helixes are folded at the N terminus, and -CCXX structure and p-loop structure are at the C-terminal. The -CCXX structure is often modified by prenylation, contributing to the location of Rab5 in the plasma membrane. P-loop consists of three parts: (1) 27–34 residues induce the hydrolysis, binding, and dissociation of GTP in Rab5, (2) 49–51 residues act as switch I, and (3) 79–81 residues act as switch II [13].

The present studies on the mutants of Rab5 focus on S34N, Q79L, A30P, G78I, N125I, N133I, D136N, and C-terminal and N-terminal truncations. Wherein, Rab5-S34N, a persistently inactive form of Rab5, is a guanylate-bound deficient mutant, and preferable to bind GDP. Overexpression of the dominant negative Rab5-S34N inhibits fusion of early endosomes and endocytosis of transferring [14]. Rab5-Q79L is a GTP enzyme-deficient mutant and can impede GTP hydrolysis, sustaining the activation of Rab5. Overexpression of Rab5-Q79L induces the fusion and expansion of endosomes and suppresses lysosome generation [15].

Rab5 transforms between the activated form GTP-Rab5 and inactivated form of GDP-Rab5. The activation of Rab5 is regulated by GEFs, and the inactivation is regulated by GAPs [16]. GEFs contain the conserved Vps9 domains [17], which may catalyze the transformation of Rab5 between GDP-Rab5 and GTP-Rab5, such as Rabex-5 [18], RME-6 [19, 20], RIN1 [21], and p85 [22–24]. GAPs regulate the activated state of Rab5, such as Rab-GAP5 [25], tuberin [26], and Armus/TBC-2 [27] (Table 1).

### 3.1. The Effectors of Rab5

Rab5 recruits the effector proteins via their GTP-dependent switch I and II to distinct subcellular compartments to regulate membrane trafficking events. The crucial effectors of Rab5 are early endosome antigen-1 (EEA1) [28, 29], rabaptin-5 [30, 31, 37], rabenosyn-5 [32, 38], APPL1/2 [33, 34], and ZFYVE21 [35] (Table 1).

EEA1, a key effector of Rab5 with a molecular weight of 162 kD, is a biomarker for early endosomes and has a parallel coiled-coil homodimer structure. EEA1 contains two binding sites for Rab5 : N-terminal C_2_H_2_ zinc finger structure and C-terminal domain [29], which can form complexes with Rab5 and specifically binds to phosphatidylinositol 3-phosphate. Phosphatidylinositol 3-phosphate further enhances the stability of GTP-Rab5, ensuring the recruitment of EEA1 to early endosomes [39]. Then, Rab5 competes with soluble NSF attachment protein receptors (SNAREs) [40] and fuses with the C-terminal of EEA1, mediating the docking of Rab5 on the membrane and regulating early endosome transport [41].

Rabaptin-5 is another Rab5 effector that plays a crucial role in membrane docking [30]. Rab5 interacts with the C-terminus of rabaptin-5 to form a complex, with its binding affinity reflecting the Rab5 activation level. The remaining structures of rabaptin-5 interact with other molecules, such as Rab4 and Rab11, to regulate the recirculation of receptors [37]. Knockdown of rabaptin-5 promotes the formation of extracellular circulating vesicles, and overexpression of rabaptin-5 exerts inhibitory effects, which reveals that rabaptin-5 maintains the balance of the receptors on the plasma membrane [31, 42].

There is close interaction among GEFs, Rab5, and Rab5 effectors. For example, after activation by Rabex-5, Rab5 recruits its effector Vps15 to interact with phosphatidylinositol 3-kinase (PI3K). Then, PI3K generates phosphatidylinositol 3-phosphate, which further recruit more effectors to interact with Rab5. Moreover, activated Rab5 interacts with its effector rabaptin-5 to form a complex. Rabaptin-5 further promotes the activity of Rabex-5 to facilitate the positive feedback from GTP-Rab5 and the binding of Rab5 to its downstream effectors [43].

## 4. The Function of Rab5 in Membrane Receptor Trafficking and Signal Transduction

**Figure 2:**
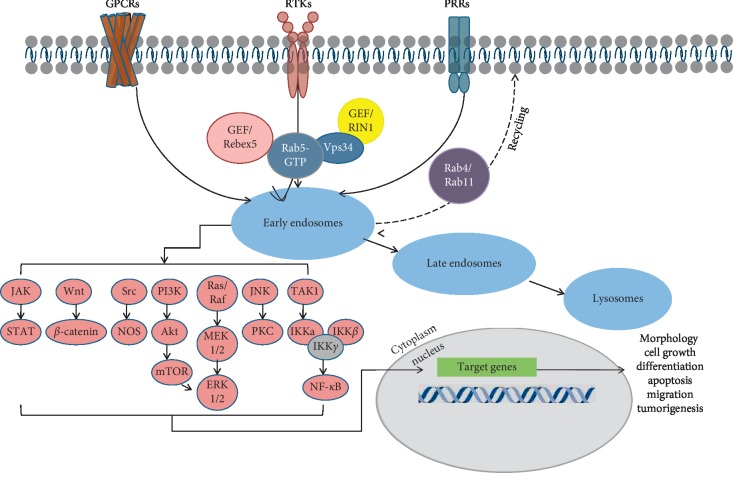
Roles of Rab5 in the internalization of receptors and signaling pathways. The receptors are sorted through the early endosome. The GTP-Rab5 recruits GEF Rabex5, which stabilizes Rab5, and Vps34 regenerates phosphatidylinositol 3-phosphate. Most of the receptor enter into the late endosomes and then degrade after reaching the lysosomes, and some recycle to the cell membrane via Rab4 and Rab11. Rab5 involves numerous signaling pathways, which may influence the cell progress and disease development (see the text for further details).

Rab5 affects the internalization and intracellular transport of receptors, such as receptor tyrosine kinases (RTKs), G protein-coupled receptors (GPCRs), and antigen recognition receptors by recruiting Rab5 effectors. The signal transduction of receptors occurs in early endosomes, further affecting gene transcription and ultimately affecting cell morphology, growth, differentiation, apoptosis, and disease development as shown in Figure 2.

### 4.1. Rab5 and RTKs

RTKs are a large superfamily of receptors that can bind with ligands and phosphorylate tyrosine residues of the target proteins through tyrosine kinase domains. They have similar structures, including the extracellular glycosylated peptides, which are responsible for binding to ligands, hydrophobic transmembrane domain, and the intracellular region with tyrosine kinase activity [44, 45]. RTKs have important physiological functions, regulating cell proliferation, cell differentiation, tumorigenesis, and tumor development [46, 47]. These receptors are categorized into several families according to the similarity of their peptide sequences and other structural characteristics, mainly including the epidermal growth factor (EGF) receptor family, platelet-derived growth factor (PDGF) receptor family, nerve growth factor receptor family, fibroblast growth factor receptor family, vascular endothelial growth factor (VEGF) receptor family, and hepatocyte growth factor receptor (c-MET) family.

Endocytosis of RTKs includes internalization, transport, sorting, and degradation [48, 49], which stimulates downstream signals and regulates cellular processes, such as cell proliferation, migration, and morphological changes. The internalization of RTKs mainly depends on clathrin. After stimulation by the ligands, cell surface invaginates and the adapter molecules recruit RTKs to clathrin-coated pits [50], which then enter into the cell. With catalyzation by dynamin [51], RTKs are transported into the cytoplasm to form clathrin vesicles and fuse with early endosomes (mainly Rab5/EEA1-positive early endosomes). Then, RTKs are transported to the late endosomes together with the early endosomes, promoting the formation of the multivesicular body. Subsequently, the multivesicular body enters into the late endosomes and finally degraded after reaching the lysosomes via the endosomal sorting complex required for transport to terminate the RTK signal [52]. RTKs, sorted through the early endosome, can be also recycled to the cell membrane via Rab4- and Rab11-positive endosomes [53].

The EGF receptor is the most widely studied molecules among RTKs. The extracellular region of the EGF receptor consists of 622 amino acid residues, which bind multiple kinds of ligands including EGF and TGF*α* [54]. In addition to the important function of Rab5 in the clathrin vesicle formation, Rab5 promotes the formation of the early endosomes by regulating vesicle fusion [55]. Knockdown of Rab5 inhibits EGF receptor internalization and trafficking, resulting in decreased EGF receptor degradation and sustained signaling transduction. In addition, Rab5-Q79L or the EGF receptor kinase inhibitor, AG1478, may inhibit the formation of Rab5-positive early endosomes, reduce the colocalization of the EGF receptor and Rab5, and further suppress endosome fusion. Rab5 GEF Rin1 restores the inhibitory effect of the AG1478 or Rab5-Q79L mutant on endosome fusion to a certain extent [56].

GEFs or interacting proteins of Rab5, such as phospholipase D (PLD), hypoxia inducible factor (HIF), neuropilin-2 (NRP2)/WDFY1 axis, and leucine-rich repeat kinase 2 (LRRK2), may also regulate the internalization, transport, and downstream signaling pathways of the EGF receptor [57]. In addition, our previous study found that *CMTM3*, a tumor suppressor gene, decreased EGF receptor expression and EGF-mediated tumorigenicity by promoting Rab5 activity in gastric cancer [58].

PLD can directly affect the upstream molecules of the EGF receptor and interact with GAP proteins, during which, Rab5 regulates EGF receptor endocytosis, clathrin vesicles formation, and finally affects EGF receptor function [31]. By downregulating the expression of Rab5 effector rabaptin-5, HIF inhibits EGF receptor degradation, resulting in prolonging EGF receptor signaling and promoting cell proliferation and survival. Pleckstrin homology (PH) domain of PLD1 may be associated with HIF and restore the decreased rabaptin-5 expression and the inhibited EGF receptor degradation [31, 59]. WDFY1, a downstream molecule of NRP2 colocalizes with EEA1 and promotes the maturation of endosomes, which affect the transport and degradation of the EGF receptor. NRP2 deletion results in a large accumulation of EEA1/Rab5 in early endosomes, downregulating late endosomes marker Rab7, delaying the maturation process of early endosomes to late endosomes, and finally inhibiting the formation of lysosomes. Moreover, NRP2/WDFY1 axis plays an important role in cancer cell endocytosis. In cancer cells, the expression of NRP2 is negatively correlated with WDFY1. NRP2 deletion leads to abnormal activation of Erk signaling pathway and causes cell death [60]. LRRK2 interacts with Rab5 to coregulate vesicle formation, during which, LRRK2 phosphorylates Thr6 of Rab5 enhances Rab5 activity and promotes EGF receptor degradation [61].

 C-MET is the receptor for HGF, which is involved in cell proliferation, differentiation, and signal transduction and regulation of cytoskeleton rearrangement. C-MET is closely associated with tumorigenesis and development of various cancers. Rab5 is also involved in the transport and signal transduction of c-MET. PTP1B interacts with c-MET, EGF, and PDGF receptors, affecting their internalization. Deletion of PTP1B promotes the phosphorylation of NSF and reduces the formation of phosphatidylinositol 3-phosphate-positive early endosomes and the activation of Rab5, resulting in inhibition of c-MET and EGF receptor transport and degradation [62]. Knockdown of NSF influences signal transduction and recirculation of c-MET, EGF receptor, integrin, and IGF-1 receptor, leading to restraining of the receptors in vesicles instead of entering the nucleus and an ultimately sustained activation of c-MET/MEK1/2 and EGF receptor/MEK1/2 signaling pathways [63, 64].

In addition, Rab5 plays a role in PDGF receptor internalization and trafficking [65]. P85, a subunit of phosphatidylinositol 3-phosphate with GAP activity [22], regulates the endosome transport, recirculation, and downstream signal activation of receptors and maintains the balance of the receptors [66]. The p85 mutant p85-R274 reverses p85 activity, inducing the accumulation of Rab5 in the cytoplasm and promoting the internalization of the PDGF receptor in a Rab5-dependent manner. Stable overexpression of p85-R274 in NIH3T3 cells reduces Rab5 activity, inhibits the degradation of the PDGF receptor, and activates downstream PI3K/Akt signaling pathway, resulting in changing in the cell morphology, promoting cell proliferation, and increase in the risk of cancer [23, 67]. However, overexpression of Rab5-S34N mutant can reverse these effect [23]. The classic Rho GTPases family member RhoD is located in early endosomes and recycling endosomes and is an interaction protein of rabankyrin-5 (a Rab5 effector) [36]. RhoD is involved in the transport of the endosome and affects PDGF receptor internalization and its downstream PLC and Akt signaling pathways [65].

Rab5 affects the internalization, trafficking, and signal transduction of the VEGF receptor and colony-stimulating factor 1 receptor. Overexpression of Rab5-Q79L in endothelial cells increases the size of early endosomes and induces the colocalization of EEA1 and VEGF receptors in endosomes, while knockdown of Rab5 enhances the activation of VEGF receptor (Y1175)/MAPK p42/44 signaling pathway [68]. Colony-stimulating factor 1 receptor colocalizes with Rab5 in macrophages. Knockdown of Rab5 inactivates p110*δ* (a catalytic subunit of Class I PI3K) and inhibits colony-stimulating factor 1 receptor downstream Akt signaling pathway, ultimately affecting the function of macrophages [69].

### 4.2. Rab5 and GPCRs

GPCR family, the largest and the most important membrane receptor superfamily in human, has more than 2000 members and is involved in virtually all life activities. The structure of GPCRs includes extracellular N-terminal domain, seven transmembrane helices (TM1-TM7) [70], intracellular C-terminal domain, three extracellular loops (ECL1-ECL3), and three or four intracellular loops (ICL1-ICL4). The amino acids of the transmembrane helical region of GPCRs are relatively conservative, while the amino acids of C-terminal, N-terminal, and loop regions are various. The abnormal expression of GPCRs may cause many diseases, such as Alzheimer's disease, Parkinson's disease, dwarfism, and color blindness, and it may affect tumorigenesis and tumor development [71].

Upon ligands stimulation, GPCRs are phosphorylated rapidly by GPCR kinases, and they bind to adapter protein *β*-arrestins to (1) inhibit the interaction of GPC receptors with G proteins, resulting in signal termination and (2) promoting endocytosis of GPC receptors, most of which are mediated by clathrin and catalyzed by dynamin [72, 73]. The endocytosis, trafficking, and functions of GPCRs are regulated by Rab GTPases. *β*-Arrestin induces the trafficking of GPCRs to the coated pits via *β*2-adaptin and clathrin [74]. After internalization, GPCRs are dephosphorylated in endosomes and then recycled to the cell membrane or stay in the early endosomes, followed by transporting into late endosomes and lysosomes for degradation [75, 76].

Rab5 is involved in the internalization and trafficking of GPCRs by regulating vesicle fusion and receptor sorting in early endosomes [77]. The transport of NK1R is regulated by Rab5, which promotes the accumulation of NK1R in the perinuclear early endosomes. Next, NK1R enters into the late endosomes and lysosomes. However, Rab5-S34N induces the retention of NK1R in early endosomes on the membrane [78]. Blocking NK1R suppresses the phosphorylation of p70S6K and 4E-BP1/2, resulting in inhibition of classical Wnt signaling pathway, which ultimately inhibits cell proliferation [79]. These findings illustrate that Rab5 not only plays a key role in the regulation of NK1R transport but also affects the related signaling pathways to make a contribution to tumorigenesis and tumor development.

Lysophosphatidic acid (LPA) is involved in metabolism, signal transduction, regulation of organ function, and is associated with inflammation [80, 81] and cancer [82, 83]. Rab5-S34N inhibits the internalization of the LPA receptor and the activation of serum response factor that is dependent on LPA [84], which further suppresses downstream signaling pathways and inhibits tumor cell motility and migration [85]. CB2 is phosphorylated via ligand stimulation [86], which promotes cell proliferation [87, 88]. Overexpression of Rab5-S34N inhibits the internalization of CB2, but has no obvious effect on CB2 recycling [89]. Leucine-rich repeat-containing G protein-coupled receptor 5 (LGR5) is involved in Wnt signaling pathway and plays an important role in a variety of tissue stem cells. After internalization, LGR5 migrates from clathrin-coated pits, enters rapidly into EEA1/Rab5-positive early endosomes, and colocalizes with Rab5. After binding with R-spondins, LGR activates Wnt/*β*-catenin signaling pathway and affects disease development [90, 91].

In addition, Rab5 colocalizes with the oxytocin receptor [92], CXCR2 [93], and other various GPCRs. The study of its mechanism will help us understand the occurrence of disease and provide new ideas for disease treatment.

### 4.3. Rab5 and Antigen Recognition Receptors

In addition to the aforementioned receptors, Rab5 is involved in the transport and signal transduction of antigen recognition receptors, such as pattern-recognition receptors (PRRs) in innate immune cells, T-cell receptor (TCR), and B-cell receptor (BCR) in adaptive immune cells.

PRRs can recognize pathogen-associated molecular patterns, which can activate a series of signaling pathways and trigger innate immune responses. PRRs include toll-like receptors (TLRs), C-type lectin receptors, NOD-like receptors, RIG-I-like receptor, and DNA-sensing molecules in the cytoplasm [94].

By binding with TLR4, lipopolysaccharide activates inflammatory-related cells and leads to inflammation [95]. The colocalization of TLR4 and Rab5 can be observed in bone marrow-derived macrophages and hematopoietic stem cells, and progenitor cells upon lipopolysaccharide stimulation [96]. Rab5 affects both TLR4 downstream NF-κB signaling and the downstream of target genes, such as *Hif-1* and *CCL2*, ultimately promoting the amplification of bone marrow-derived multifunctional hematopoietic stem cells [97].

Mannose and scavenger receptors are macrophage surface receptors, which participate in pathogen recognition, antigens presentation, and maintain homeostasis [98, 99]. Both mannose and scavenger receptors colocalize with Rab5 [100–102]. IL4/PGE2 stimulation significantly upregulates the expression of the mannose receptor, Rab5, and Rab5 GEF Rin1 in mouse bone marrow-derived macrophages, eventually promoting phagocytosis of mouse bone marrow-derived macrophages [103].

In addition, Rab5 is involved in the transport and signal transduction of TCR and BCR. TCR forms complex with Rab5 in early endosomes and accumulates in Rab5-positive early endosomes [104]. The reduced activity of Rab5 inhibits TCR degradation and enhances TCR signaling pathways. In mouse Th2 cells, knockdown of Rab5 selectively affects TCR downstream signaling and promotes the production of the corresponding cytokines [105]. It was reported that the number of CD4^+^CD8^+^ thymocytes is obviously reduced in T-cell-specific Rab5-N133I transgenic mice, suggesting that Rab5 plays a key role in TCR transport and signal transduction [106].

The internalization of BCR and BCR-mediated signal transduction establish a series of checkpoints in B cells to ensure B-cell maturation, BCR receptor formation, and humoral immune response generation [107]. Upon antigen stimulation, BCR transmits signals to extend cell morphology, and the clathrin-coated pits are generated in BCR-antigen clusters [108]. Rab5 promotes the formation of early endosomes in the internalized vesicle fusion and triggers Erk, p38, JNK, and Akt signal to affect further the life processes of cells.

Overall, there are two methods we suggest to treat diseases according to the current Rab5-related studies. First, a direct interaction with Rab5, such as Rab5-targeted therapies, that can transform the activation of Rab5 and influence receptor internalization and trafficking, leading to physiological changes of patients. Second, an indirect way to regulate Rab5 by influencing Rab5 effectors, GEFs, or GAPs is another potential strategy via influencing the aberrant expression, internalization, trafficking, and degradation of receptors. At present, the clinical cancer treatment, for instance, is difficult to achieve satisfactory prognosis of cancer patients because of the high recurrence and metastasis tendency after surgery, and the resistance to radiotherapy and chemotherapy. Thus, regulation of the Rab5 strategy may relieve the cancer patient's distress and provide us a novel idea for cancer therapy.

## 5. Conclusions

Rab5 is a key factor in regulating early endocytosis. Rab5, recruits its effectors to early endosomes, is involved in the transport of endosomes, and affects membrane receptor internalization, trafficking, and related signaling pathways, which contribute to gene transcription and the biological processes of cells. The mutation of Rab5 can cause abnormal cell morphology and function, suggesting that the structure of Rab5 is closely related to its function and occurrence and development of diseases. However, the mechanisms of Rab5 in diseases are not fully understood and need further investigation.

In summary, the study of Rab5 will help us understand the regulation mechanisms of receptor internalization and trafficking and provide new ideas and targets for the treatment of related diseases.

## Figures and Tables

**Table 1:** Summary of Rab5 regulator and effectors.

			Key structures	Functions	References
Regulators	GEFs	Rabex-5	Ubiquitin-binding domain, E3 ubiquitin ligase domain	Activation of Rab5 GTPases during endocytosis	[18]
		RME-6	Vps9-domain	Regulation of clathrin-coated vesicle uncoating and delivery of endocytic cargo to early endosomes	[19, 20]
		RIN1	Proline-rich domain, tyrosine 36	Internalization, trafficking and degradation of activated receptors, cytoskeleton remodeling	[21]
		p85	C-terminal and N-terminal domains	Activation of Rab5 GTPases during endocytosis, migration of cancer cells	[22–24]
	GAPs	Rab-GAP5	Tre2/Bub2/Cdc16 domain	Inactivation of Rab5 GTPases during endocytosis and trafficking	[25]
		Tuberin	C-terminal domain	Inactivation of Rab5 GTPases during endocytosis and trafficking	[26]
		Armus/TBC-2	PH domain	Inactivation of Rab5 to promote Rab5 to Rab7 conversion during endosome maturation	[27]

Effectors		EEA1	C-terminal and N-terminal domains, C_2_H_2_ zinc finger domain	Fusion, docking and sorting of the early endosome	[28, 29]
		Rabaptin-5	C-terminal domain	Fusion, docking and sorting of the early endosome	[30, 31]
		Rabenosyn-5	N-terminal domain, C_2_H_2_ zinc finger domain, and FYVE finger domain	Regulation of macropinocytosis, initiation of tubular endocytosis and surface flattening	[32, 33]
		APPL1/2	PH domain, PTB domain, and leucine zipper motif	Stable cargo-sorting compartments, membrane traffic/signaling, cell proliferation	[33, 34]
		ZFYVE21	FYVE-finger domain	Phosphoinositide remodeling of early endosome membranes to mediate signal activation and tissue inflammation	[35]
		Rabankyrin-5	FYVE finger domain, ankyrin repeats	Formation of endosomes and remodeling of the apical plasma membrane	[36]
